# A rare large cell variant of small-cell carcinoma of the ovary, hypercalcemic type in a postmenopausal woman: Case report

**DOI:** 10.1097/MD.0000000000046423

**Published:** 2025-12-19

**Authors:** Simeng Liu, Shuping Che, Cuicui Jin, Yuhui Deng, Deli Zhao

**Affiliations:** a Department of Radiology, Sixth Affiliated Hospital of Harbin Medical University, Harbin, China; b Department of Radiology, First Affiliated Hospital of Harbin Medical University, Harbin, China.

**Keywords:** age, large cell variant, MRI, SCCOHT, SMARCA4 mutation

## Abstract

**Rationale:**

Small-cell carcinoma of the ovary, hypercalcemic type (SCCOHT) is an extremely rare and aggressive ovarian malignancy associated with SMARCA4 mutation, accounting for less than 0.01% of ovarian tumors. It predominantly affects young females and is characterized by poor prognosis. Here, we report a unique case of SCCOHT with a special pathological classification of large cell variant type in a 59-year-old postmenopausal woman, an age that is rare for this disease.

**Patient concerns:**

A 59-year-old female patient presented with lower abdominal pain. A hard and restricted mass was palpated at the back of the uterus during pelvic physical examination. MRI examination showed a huge solid mixed-signal mass shadow in the right adnexal area, with a size of 10 × 7 × 7 cm.

**Diagnoses:**

Postoperative histopathological and immunohistochemical analyses demonstrated abundant large cells under microscopic examination and a loss of BRG1 protein expression, leading to the diagnosis of large cell variant of SCCOHT in right ovary. Genetic testing confirmed a mutation in the SMARCA4 gene (exon 6, c.1103del p.[Q368Rfs*43]).

**Interventions:**

The patient underwent pelvic tumor cytoreductive surgery and pelvic adhesion release surgery, and the implanted lesions in the pelvic cavity were resected during the operation.

**Outcomes:**

The patient underwent extensive surgical resection and completed 6 cycles of TC chemotherapy (paclitaxel + carboplatin). Despite the postoperative pathological stage reached Stage II, during the 18-month follow-up after the operation, no evidence of tumor progression or recurrence was observed.

**Lessons:**

SCCOHT is a highly malignant tumor associated with a generally poor prognosis, particularly for tumors at stage II or higher. Nevertheless, the favorable prognosis observed in this case suggests that the traditional understanding may require reevaluation. Whether there is a correlation between the age of onset of SCCOHT and the prognosis remains to be elucidated through systematic clinical research in the future.

## 1. Introduction

Small-cell carcinoma of the ovary, hypercalcemic type (SCCOHT) is a rare and highly aggressive ovarian malignancy associated with SMARCA4 mutations. The incidence of SCCOHT is less than 0.01% of ovarian tumors.^[1,2]^ It occurs predominantly in young women (range 0–55 years, median age about 24 years), and the prognosis is extremely poor.^[3,4]^ This article describes a 59-year-old patient with SCCOHT, which is the rare age group reported in the public literature. In addition, the pathological classification of this case is a special type of large cell variant, which has not been widely reported before. More importantly, this patient was treated with standard therapy and followed up for up to 18 months without tumor progression or recurrence, which subverted the previous understanding that SCCOHT had a very poor prognosis.^[5]^ We hope that this case report will provide a new perspective and reference for clinical research of SCCOHT and further explore pathogenesis, diagnostic methods, and treatment strategies of SCCOHT to improve prognosis of patients.

## 2. Case presentation

### 2.1. Clinical information

The patient was a 59-year-old postmenopausal woman who came to our hospital in June 2023 due to lower abdominal pain for 15 days. Current history is that half a month ago, the patient presented with lower abdominal pain without obvious cause, especially in the right lower abdomen, accompanied by low back pain. The pain was aggravated when walking. The patient had a past history of menopause at the age of 54, and her obstetric history was G2P1A1L1. Her blood pressure on admission was 148/87 mm Hg, but she had no previous history of hypertension. After a physical examination by a gynecological oncologist, uterus was in the anterior position with good mobility, and a non-active mass was palpated in the pelvic cavity behind the uterus. On admission, the laboratory indexes, mainly including serum electrolytes (Ca) and tumor markers (alpha-fetoprotein, carbohydrate antigen 125, carbohydrate antigen 19-9, carcinoembryonic antigen, human epididymis protein 4, risk of ovarian malignancy algorithm index), were all within the normal range (Table 1).

**Table 1:** Blood biochemical indicators.

Project name	Test result	Reference range	Unit
Ca	2.41	2.11–2.52	mmol/L
AFP	1.85	0–7	ng/mL
CA 125	16.72	0–35	U/mL
CA19-9	17.46	0–30	U/mL
CEA	2.22	0–4.5	ng/mL
HE4	38.40	0–74.31	pmol/L
ROMA-After	9.67	0–29.9	%

AFP = alpha-fetoprotein, Ca = calcium, CA 125 = carbohydrate antigen 125, CA 19-9 = carbohydrate antigen 19-9, CEA = carcinoembryonic antigen, HE4 = human epididymis protein 4, ROMA-After = risk of ovarian malignancy algorithm – postmenopausal.

### 2.2. Imaging findings

**Figure 1.**
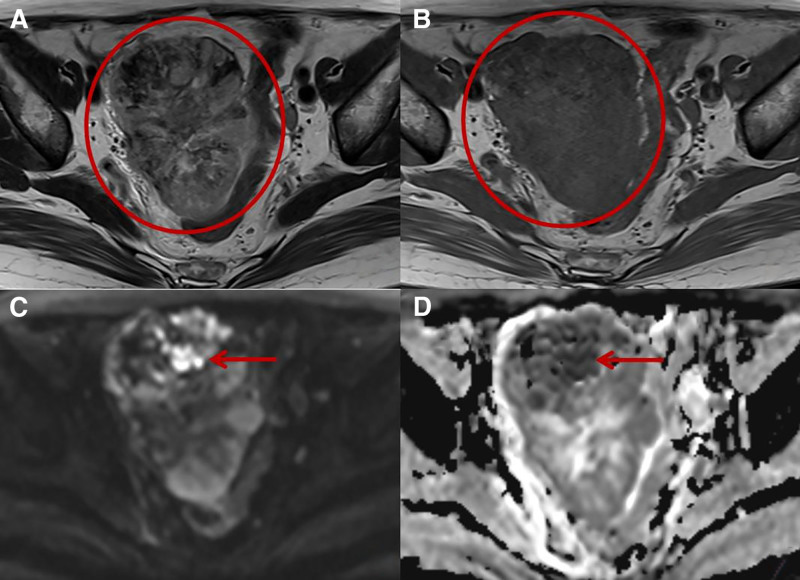
Plain MRI scan of the patient’s pelvic cavity. Axial T2WI (A) and T1WI (B) showed mixed signal solid space-occupying shadows in the right adaccessory area, with a size of 10 × 7 × 7 cm; DWI (C) and ADC (D) (b = 800 s/mm^2^) showed significantly limited local diffusion within the tumor, with an ADC value of approximately 0.447 × 10^−3^ s/mm^2^.

After admission, the patient underwent pelvic MRI examination, which indicated a mixed-signal mass in the right adnexal area, suggesting a possible malignant tumor (cystadenocarcinoma) of the right ovary. Other diagnoses included uterine fibroids, cervical nabothian cysts, and pelvic effusion (Fig. 1). The upper abdominal MRI showed multiple cystic lesions in the liver, which were considered as simple cysts, and small amount of perihepatic effusion was also found. Chest CT showed a pure ground-glass nodule in the upper lobe of the right lung, with a diameter of approximately 5 mm (classified as Lung-RADS 3).

### 2.3. Surgery

Subsequently, the case was discussed at the multidisciplinary team meeting of our institution’s gynecological oncology department. The initial clinical judgment is stage II. Pelvic tumor cytoreductive surgery and pelvic adhesion release surgery are planned, and intraoperative rapid frozen pathological examination is also scheduled. During the operation, it was found that the volume of the right ovary had increased, and the size of the mass was approximately 10 × 7.5 × 5 cm. Multiple granular or nodular protrusions can be seen on the surface of the left ovary, beside the right fallopian tube, and in the uterorectal pouch. Intraoperative observation revealed that the appendix and the local greater omentum might have been invaded, so they were removed and sent for examination. Frozen pathology during surgery showed malignant tumor with necrosis in the right adnexa, and the possibility of poorly differentiated carcinoma was high. The rest of the tumors were further classified by paraffin section and immunohistochemistry.

### 2.4. Pathological and genetic results

**Figure 2.**
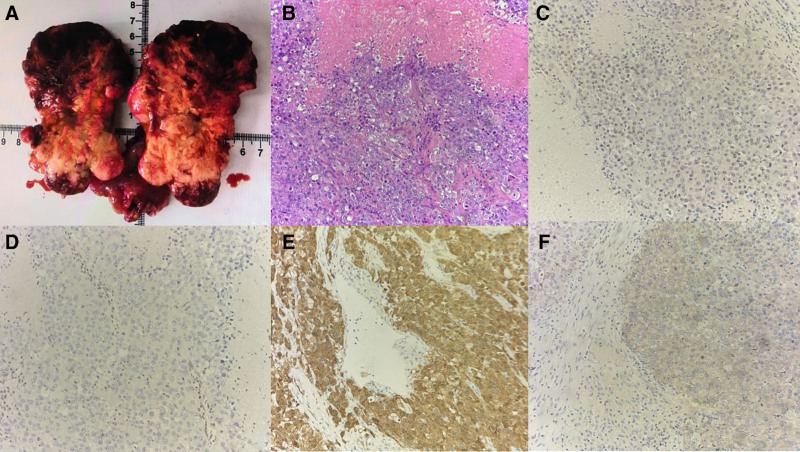
Various indicators under the microscope and immunohistochemistry pointed to large cell variant SCCOHT. (A) Gross profile of the tumor; (B) HE staining (×100); (C) Immunohistochemical result (×200) showed BRG1 (−); (D) Immunohistochemical result (×200) showed WT-1 (part +); (E) Immunohistochemical result (×200) showed P16 (+); (F) Immunohistochemical result (×200) showed EMA (part +). SCCOHT = small-cell carcinoma of the ovary, hypercalcemic type.

According to postoperative histopathology and immunohistochemistry, the patient was diagnosed as right ovarian SCCOHT (large cell variant), vascular tumor embolus (+), and fallopian tube carcinoma (+). Tumor tissue was found near the uterine rectal fossa and right fallopian tube. Lymph nodes (−) in each group. No significant changes were observed in the appendix and greater omentum tissues. The final pathological International Federation of Gynecology and Obstetrics stage was Stage II. Immunohistochemical (part key indicators): BRG1 (−), EMA (part +), WT-1 (part +), p16 (+), p53 (nonsense mutation among mutant type), ER (−), PR (−), and Ki-67 (90%) (Fig. 2). Remark: Since SCCOHT (large cell variant) is very rare, morphology and absence of BRG1 expression in this case support the diagnosis, and it is recommended to do genetic testing for further confirmation. One month after surgery, genetic testing revealed a positive SMARCA4 mutation (Table 2).

**Table 2:** Genetic testing results.

Gene	Test result	Abundance/copy number	Variation classification	Clinical significance
NF1	exon50 c.7348C > T p.(R2450*)	31.80%	II类	Bimetinib (sensitive, Grade C)
PIK3CA	exon2 c.333G > T p.(K111N)	28.03%	II类	Apelix + fulvestrant (Sensitive, Grade C)
PIK3CA	exon10 c.1625A > C p.(E542A)	27.33%	II类	Apelix + fulvestrant (Sensitive, Grade C)
POLE	exon13 c.1231G > T p.(V411L)	29.15%	II类	PD-1 inhibitor (sensitive, Grade C)
TP53	exon6 c.637C > T p.(R213*)	57.42%	II类	Adavosertib + carboplatin + paclitaxel (sensitive, Grade C)
CREBBP	exon26 c.4349A > G p.(Y1450C)	31.97%	II类	C646 (Sensitive, Class D)
EGFR	exon19 c.2258C > T p.(P753L)	27.76%	II类	Afatinib (sensitive, Grade D)
SMARCA4	exon6 c.1103del p.(Q368Rfs*43)	56.16%	II类	Alisertib (Sensitive, Grade D)

### 2.5. Prognosis

Due to the rarity of SCCOHT, there is currently no standardized postoperative chemotherapy regimen internationally. Therefore, in accordance with the National Comprehensive Cancer Network guidelines for the diagnosis and treatment of ovarian cancer,^[6]^ a conventional first-line chemotherapy regimen of paclitaxel + carboplatin is adopted. The patient was treated with TC (paclitaxel 210 mg + carboplatin 600 mg) regimen for 6 courses after surgery and was followed up for 18 months (Table 3). Surprisingly, both imaging and laboratory examination did not show disease progression or recurrence.

**Table 3:** Postoperative follow-up summary.

Follow-up time	Therapeutic measures	Imaging examination (MRI/US/CT)			Tumor markers (AFP, CA 125, CA19-9, CEA, HE4)
		Full abdominal MRI	Pelvic US	Chest CT	
Postoperative 3 mo	Postoperative adjuvant chemotherapy, Course 2	Liver cystic lesion without significant change; no abnormalities in the upper abdomen; postoperative changes in the pelvis, no abnormalities found	/	Right upper lobe nodule, diameter 5 mm; no abnormalities in the remaining bilateral lungs	Within normal range
Postoperative 6 mo	Postoperative adjuvant chemotherapy, Course 4	Liver cystic lesion without significant change; no abnormalities in the upper abdomen; postoperative changes in the pelvis, no abnormalities found	/	Right upper lobe nodule, diameter 5 mm; no abnormalities in the remaining bilateral lungs	Within normal range
Postoperative 9 mo	Postoperative adjuvant chemotherapy, Course 6	Liver cystic lesion without significant change; no abnormalities in the upper abdomen; postoperative changes in the pelvis, no abnormalities found	/	Right upper lobe nodule, diameter 5 mm; no abnormalities in the remaining bilateral lungs	Within normal range
Postoperative 12 mo	/	/	Postoperative changes in the pelvis, no abnormalities found	Right upper lobe nodule, diameter 5 mm; no abnormalities in the remaining bilateral lungs	Within normal range
Postoperative 15 mo	/	/	Postoperative changes in the pelvis, no abnormalities found	Right upper lobe nodule, diameter 5 mm; no abnormalities in the remaining bilateral lungs	Within normal range
Postoperative 18 mo	/	/	Postoperative changes in the pelvis, no abnormalities found	Right upper lobe nodule, diameter 5 mm; no abnormalities in the remaining bilateral lungs	Within normal range

AFP = alpha-fetoprotein, CA 125 = carbohydrate antigen 125, CA 19-9 = carbohydrate antigen 19-9, CEA = carcinoembryonic antigen, HE4 = human epididymis protein 4.

### 2.6. Case summary

A 59-year-old postmenopausal woman presented with lower abdominal pain and a palpable mass. MRI revealed a large mixed-signal mass in the right adnexal area, measuring 10 × 7 × 7 cm. The patient underwent pelvic mass resection, and intraoperative cryo-pathological examination indicated a malignant tumor. Postoperative histopathology and immunohistochemistry confirmed the diagnosis of SCCOHT with a SMARCA4 mutation (exon 6, c.1103del p.[Q368Rfs*43[). The patient completed 6 cycles of TC chemotherapy (paclitaxel + carboplatin) after the operation. During the 18-month follow-up, no evidence of tumor progression or recurrence was observed (Table 4).

**Table 4:** Case summary.

Event	Details
Patient information	59-year-old postmenopausal woman with lower abdominal pain and palpable mass
Imaging findings	MRI: large mixed-signal mass in right adnexal area (10 × 7 × 7 cm)
Surgical procedure	Pelvic tumor cytoreductive surgery and pelvic adhesion release surgery
Postoperative diagnosis	SCCOHT with SMARCA4 mutation (exon 6, c.1103del p.(Q368Rfs*43))
Treatment	6 cycles of TC chemotherapy
Follow-up	18 mo; no evidence of tumor progression or recurrence

SCCOHT = small-cell carcinoma of the ovary, hypercalcemic type, TC = paclitaxel + carboplatin.

## 3. Discussion

### 3.1. Molecular pathology

SCCOHT is a rare and highly aggressive type of ovarian cancer. Due to unclear histological origin, it has been classified as a hybrid ovarian tumor by World Health Organization in 2020.^[7]^ Witkowski et al^[8]^ found that SMARCA4 mutation played a key role in the development and progression of SCCOHT. Recent molecular analyses have shown that SCCOHT is a monogenic disease associated with SMARCA4 inactivation mutation. SMARCA4 is a member of SWI/SNF chromatin remodeling complex, which has anti-cancer function.^[9]^ SMARCA4 mutations are detected in > 90% of SCCOHT cases, as compared with 0.4% of other primary ovarian tumors.^[10]^ In this case, SMARCA4 gene mutation was detected as a frameshift mutation, resulting in loss of protein function, which further confirmed the pathological diagnosis.

The histologic features of SCCOHT are characterized by uniform small cells arranged in a variety of structures.^[11]^ However, a rare large cell variant was observed in present case, which presents glassy eosinophilic cytoplasm and eccentric large, pale nucleus under the microscope. This is different from the typical small cell morphology of SCCOHT. Studies have reported that the prognosis of SCCOHT patients with large cell components is significantly worse.^[12]^ However, the loss of SMARCA4-encoded BRG1 protein expression in the immunohistochemical staining has become the key information for pathological diagnosis. In addition, immunohistochemical staining also shows partial positivity for EMA, WT-1 and positivity for P16. The expression of these markers is helpful in the differential diagnosis from other ovarian tumors.^[13]^

### 3.2. Clinical and imaging findings

Currently, no more than 500 cases of SCCOHT have been reported in the literature worldwide.^[14]^ The most common age of onset is in young women under 40 years old, and the average age of diagnosis is 24 years old.^[3,15]^ The average age of onset of SCCOHT in 47 patients reported by MD Anderson Cancer Center was 30 years.^[1]^ The patient in this case is a 59-year-old postmenopausal woman, which is different from the common young-onset population of SCCOHT. At the same time, we observed that the better prognosis of this patient was different from previous reports. In Young et al^[16]^ large case cohort study on SCCOHT, possible favorable prognostic features include low-level International Federation of Gynecology and Obstetrics stage, age > 30 years, normal preoperative calcium level, tumor size of up to 10 cm, and absence of large cells. Therefore, we speculate that the relatively good prognosis of this patient may be related to the patient’s age and postmenopausal status to some extent. This suggests that the clinical behavior of SCCOHT might vary with age and hormonal status, which warrants further investigation. At the same time, the patient’s preoperative blood calcium level was normal, and the longest diameter of the tumor reaching 10 cm also suggested that the prognosis might be good. The most common symptom of SCCOHT is abdominal pain, followed by obvious mass, enlargement of waist circumference, vomiting, etc.^[17]^ The main symptom of the patient in this study was lower abdominal pain accompanied by lumbago, which may be related to the compression of surrounding tissues by the growth of tumor.

Lu et al^[18]^ analyzed nearly 400 cases of SCCOHT and found that the tumor diameter ranged from 6 to 30 cm, with an average diameter of 15.3 cm. Most of the tumors were unilateral, and about 66% of the cases occurred in the right ovary, which was consistent with the origin of this case. The imaging findings of this patient were consistent with the typical features of SCCOHT, which was a large solid tumor in the unilateral adnceal region. However, due to the rare nature of SCCOHT, it is challenging to differentiate it from other ovarian tumors such as dysgerminoma, yolk sac tumor, and granular cell tumor on imaging.^[19]^ But it can be differentiated through laboratory indicators (such as human chorionic gonadotropin, alpha-fetoprotein, sex hormone levels) and immunohistochemical indicators (such as SALL4, which is specifically expressed in dysgerminoma and yolk sac tumors).^[20]^ In addition, considering the aggressive nature of SCCOHT, imaging studies should also focus on the presence of lymph node metastasis or other signs of distant metastasis.

### 3.3. Treatment and prognosis

Due to the rarity of SCCOHT, there is no standardized treatment regimen, but a comprehensive treatment mode combining surgical resection of primary tumor with adjuvant chemotherapy is usually adopted.^[21]^ Surgery plays a key role in the management of SCCOHT, especially by removing as much of the tumor as possible to reduce its burden.^[22]^ In this case, the patient underwent extended pelvic resection combined with postoperative TC chemotherapy regimen and achieved a relatively good postoperative survival period. Although traditional chemotherapy can achieve significant results in some cancers, many patients still face the problems of drug resistance and recurrence. SCCOHT is a malignant tumor with obvious heterogeneity and high invasiveness, and traditional single treatment regimens are difficult to achieve ideal results. With the development of gene sequencing technology and the in-depth understanding of tumor molecular mechanisms, new therapeutic targets are constantly being discovered, providing the possibility for the development of systemic treatment plans. Emerging therapies for SCCOHT are rapidly moving from bench to bedside: EZH2 inhibitors (GSK126, EPZ-6438) exploit SMARCA4 loss to reactivate tumor-suppressive chromatin programs; the multi-kinase inhibitor ponatinib, identified by high-throughput screens, suppresses FGFR/PDGFR-driven signaling in SMARCA4-deficient cells; meanwhile, immune-checkpoint blockade (PD-1/PD-L1 inhibitors) is being combined with anti-angiogenic agents and CDK4/6 inhibitors.^[23–25]^ In addition, immune checkpoint inhibitors have shown great potential in treating various cancers, including ovarian cancer. The prognostic significance of various biomarkers, such as the neutrophil-to-eosinophil ratio and the Royal Marsden Hospital score, has been explored in recent studies, providing valuable insights into patient stratification and treatment planning.^[26–30]^ Although these strategies offer compelling biological rationale and early efficacy signals, large-scale prospective trials are still required to confirm their true benefit and optimal sequencing.

The overall prognosis of SCCOHT is poor, with a survival rate of only 30% at early diagnosis.^[8]^ In the large case report of 150 patients with SCCOHT, Young et al^[16]^ showed a mean 6-year disease-free survival of 33% for stage I patients and a 5-year survival rate of 6.5% for stage II to IV patients. In this case, the postoperative pathology confirmed that the patient had multiple metastases in pelvic organs outside the ovary, and the pathological stage was Stage II, indicating a poor prognosis. However, in this case, the conventional first-line chemotherapy regimen for ovarian cancer (TC therapy) unexpectedly demonstrated a good therapeutic effect. The successful treatment outcome in this case, despite the advanced pathological stage, suggests that the conventional TC chemotherapy regimen might be more effective than previously thought for SCCOHT, at least in some cases. This highlights the need for individualized treatment approaches and the importance of closely monitoring patients’ responses to therapy. Nevertheless, given the overall poor prognosis of patients with SCCOHT, with a very low 5-year survival rate, long-term close clinical follow-up is still required for this patient.

## 4. Conclusion

In summary, we report a rare case of SCCOHT (large cell variant) in a postmenopausal woman, highlight its unique pathological and clinical features, and analyze the molecular pathology, clinical and imaging features, treatment, and prognosis of this disease in detail. We aim to improve the understanding of this rare disease and provide a valuable reference for clinical practice. The analysis of the relevant factors influencing the prognosis of SCCOHT in the future still requires in-depth research through a larger number of cases.

## Author contributions

**Conceptualization:** Simeng Liu.

**Data curation:** Simeng Liu.

**Formal analysis:** Simeng Liu, Cuicui Jin.

**Funding acquisition:** Yuhui Deng, Deli Zhao.

**Investigation:** Shuping Che.

**Methodology:** Simeng Liu.

**Resources:** Shuping Che, Cuicui Jin.

**Supervision:** Deli Zhao.

**Validation:** Yuhui Deng.

**Visualization:** Yuhui Deng.

**Writing – original draft:** Simeng Liu.

**Writing – review & editing:** Deli Zhao.
